# A C_21_-Steroidal Glycoside from *Cynanchum atratum* Attenuates Concanavalin A-Induced Liver Injury in Mice

**DOI:** 10.3390/molecules24061087

**Published:** 2019-03-19

**Authors:** Jian Yang, Bin Wang, Chao-feng Zhang, Xiang-hong Xu, Mian Zhang

**Affiliations:** School of Traditional Chinese Pharmacy, China Pharmaceutical University, Nanjing 211198, China; yangjian_132@163.com (J.Y.); wangbin9077@163.com (B.W.); njchaofeng@126.com (C.-f.Z.); xuxh168@126.com (X.-h.X.)

**Keywords:** Cynatratoside A, autoimmune hepatitis, spleen, T lymphocyte, apoptosis, *Cynanchum atratum*

## Abstract

Cynatratoside A (CyA) is a C_21_ Steroidal glycoside with pregnane skeleton isolated from the root of *Cynanchum atratum* Bunge (Asclepiadaceae). This study aimed to investigate the effects of CyA on concanavalin A (Con A)-induced autoimmune hepatitis (AIH) and the underlying mechanism. CyA was orally administered to mice at 10 and 40 mg/kg 8 h before and 1 h after Con A treatment. The effects of CyA on Con A-induced spleen and liver in mice were assessed via histopathological changes, T lymphocyte amounts and the expressions of IL-1β and ICAM-1. Con A-induced L-02 hepatocytes were used to evaluate whether CyA (0.1–10 μM) can directly protect hepatocytes from cytotoxicity and the possible mechanism. The results revealed that CyA treatment could significantly improve the histopathological changes of spleen and liver, reduce the proliferation of splenic T lymphocytes, and decrease the expressions of IL-1β and ICAM-1 in liver. The experiment in vitro showed that CyA inhibited Con A-induced hepatotoxicity in a concentration-dependent manner. CyA (10 μM) significantly increased/decreased the expression of Bcl-2/Bax and reduced the levels of cleaved caspases-9 and -3. Our study demonstrated for the first time that CyA has a significant protective effect on Con A-induced AIH by inhibiting the activation and adhesion of T lymphocytes and blocking hepatocyte apoptosis.

## 1. Introduction

The liver is the largest digestive gland and metabolic organ in the human body. It also has the functions of detoxification, immunity and hematopoiesis [1,2]. The liver is a vital organ and supports almost every other organ in the body. More and more evidences have shown that the liver plays an important role in immune response, immune regulation and immune tolerance [3,4,5]. However, the liver is also prone to many diseases, the most common of which is hepatitis [6,7]. Although viral hepatitis is the most common, non-viral factors such as autoimmunity can also cause hepatitis. Autoimmune hepatitis (AIH) is an autoimmune disease of the liver and occurs when the body’s immune system attacks its own hepatocytes. It is a progressive liver disease with unknown etiology and poor prognosis, and can rapidly develop to cirrhosis and liver failure in severe cases [7,8,9]. Although immunosuppressive therapy is standard, most immunosuppressants have toxic or serious side effects [7]. The development of immunosuppressive agents with high efficiency and low toxicity is an urgent and challenging task.

Recently, mounting drug researches have focused on folk medicines in order to find effective and low toxicity lead compounds for drug development. *Cynanchum atratum* Bunge (Asclepiadaceae) is a perennial herbaceous plant native to East Asia and widely distributed in China [10,11]. Its dried root, known as Baiwei in Chinese, is one of traditional Chinese medicines with the functions of clearing internal heat to cool blood and cure furuncles and promoting diuresis to relieve stranguria [12,13]. The meridian tropism of Baiwei is stomach, liver and kidney meridians. Pharmacological studies showed that Baiwei has anti-tumor, immune regulation, anti-virus and anti-inflammatory activities [11,12,13,14,15,16]. C_21_ steroidal glycosides are the main active and characteristic constituents of Baiwei and most of them have significant immunosuppressive effects [15,17,18,19]. Cynatratoside A (CyA) is one of C_21_ steroidal glycosides isolated from Baiwei. Our early studies have shown that CyA inhibited splenic T lymphocyte proliferation induced by concanavalin A (Con A) with an IC_50_ (half maximal inhibitory concentration) value of 10.9 μM [15]. This result indicated that CyA had immunosuppressive activity and might be a potential candidate for the treatment of AIH.

So far, Con A-induced liver injury in mice has been regarded as an ideal model for AIH research, as it is essentially similar to the pathogenesis and pathological changes of patients [20,21]. Therefore, we studied the effect of CyA on Con A-induced liver/hepatocyte injury and its possible mechanism.

## 2. Results

### 2.1. CyA Inhibits Con A-Induced Pathological Changes of Spleen in Mice

**Figure 1:**
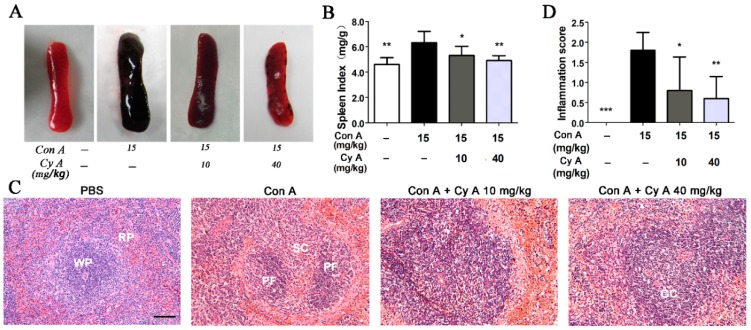
CyA alleviated pathological changes of spleen in Con A-induced mice. Mice were injected with 15 mg/kg Con A via caudal vein and orally administrated with 10 or 40 mg/kg CyA 8 h before and 1 h after Con A treatment. Splenic injury was assessed by (**A**) morphology; (**B**) spleen index (*n* = 5); (**C**) H&E stained spleen section (bar = 100 μm) and (**D**) inflammatory score (*n* = 5). GC, germinal center; PF, primary follicle; RP, red pulp; SC, splenic corpuscle; WP, white pulp. Data were expressed as mean ± SD. * *p* < 0.05, ** *p* < 0.01 and *** *p* < 0.001, versus the model group.

The spleen, containing 25% circulating T lymphocytes, is the largest immune organ and the immune center of the body. It directly participates in cellular immunity and regulates the distribution of T lymphocyte subsets in peripheral blood [22,23]. Although AIH is closely associated with the spleen, pathological changes of spleen during autoimmunity have not been reported. Therefore, the morphological and pathological changes of spleen in Con A-injured mice were observed in this study. Compared with the control group (Figure 1A,B), the spleen in the model mice was black red, fragile and swollen, and had a significant high spleen index (*p* < 0.01). CyA treatment at 10 and 40 mg/kg could ameliorate splenic pathologic status and significantly decrease the spleen index (*p* < 0.05/0.01 for 10/40 mg/kg). Histopathological changes in spleen tissue of mice were evaluated with H&E staining. The results (Figure 1C) showed that the spleen of the control group had normal white and red pulps, no congestion and inflammatory cell infiltration. The pathological changes of Con A-injured spleens were obvious, including disorder of white pulp structure, vacuolar degeneration or even atrophy or necrosis of splenic corpuscles, red pulp congestion, splenic sinus dilatation and inflammatory cell infiltration. CyA treatment could improve the structure destruction of white pulp, relieve vacuolar degeneration of splenic corpuscles and congestion of red pulp, and decrease the infiltration of inflammatory cells (Figure 1C). The curative effect of high-dose group was better than that of low-dose group. Correspondingly, the inflammation scores at both doses were significant lowered compared to the model group (*p* < 0.05/0.01 for 10/40 mg/kg) (Figure 1D).

### 2.2. CyA Suppresses T Lymphocyte Proliferation in the Spleen of Con A-Induced Mice

**Figure 2:**
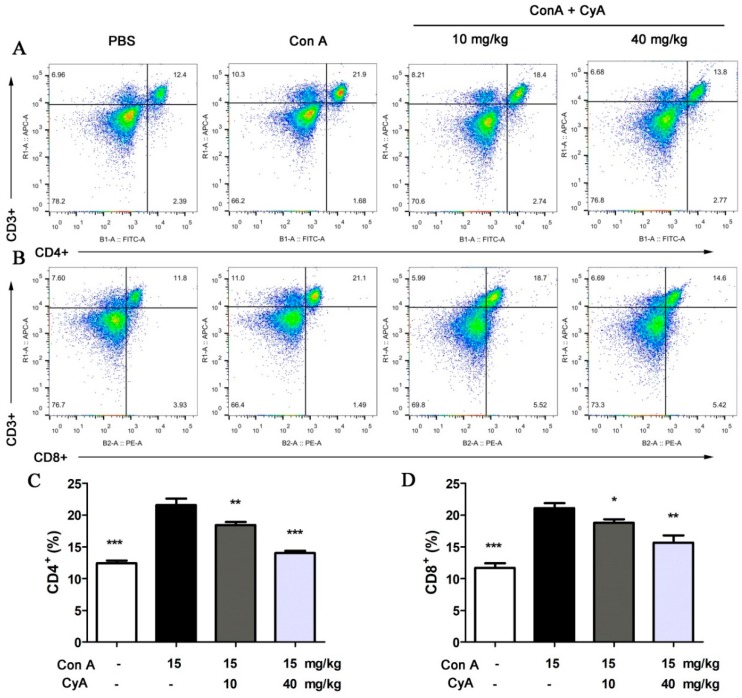
CyA inhibited T lymphocyte activation in the spleen of Con A-injured mice. Mice were treated in the same way as described in Figure 1. The spleen was removed and splenocytes were isolated and stained with anti-mouse CD3ε, FITC anti-mouse CD4 or PE anti-mouse CD8a antibodies, and detected by flow cytometer. The results shown in (**A**,**B**) are representative flow cytogram for CD4^+^ and CD8^+^ T lymphocytes of three independent experiments. The data in (**C**,**D**) are expressed as mean ± SD from the three independent experiments. * *p* < 0.05, ** *p* < 0.01 and *** *p* < 0.001, versus the model group.

It is believed that AIH is mainly associated with the CD4^+^ helper T cells, which mediate the release of inflammatory cytokines [20]. The CD8^+^ cytotoxic T cells are also involved in AIH, although they are not a major factor [24,25]. Our previous experiments in vitro demonstrated that CyA could significantly inhibit Con A (5 μg/mL)-induced proliferation of splenic T lymphocytes, with an IC_50_ value of 10.9 μM and a CC_50_ (median cytotoxic concentration) value > 80 μM [15]. In this study, the amount of T lymphocytes, including CD4^+^ and CD8^+^ T cells, in spleen tissue of mice was analyzed by flow cytometry. As shown in Figure 2A,C, the number of CD4^+^ T cells in Con A-injured spleen increased significantly from 12.5% (the control) to 21.6% (the model). Treatment with CyA significantly decreased the CD4^+^ T cells from 21.6% (the model) to 18.4% (10 mg/kg, *p* < 0.01) or 14.0% (40 mg/kg, *p* < 0.001). Similarly, the CD8^+^ T cells in Con A-injured spleen were increased from 11.7% to 21.1%, and CyA treatment decreased them from 21.1% to 18.8% (10 mg/kg, *p* < 0.05) or 15.6% (40 mg/kg, *p* < 0.01) (Figure 2B,D). These results indicated that splenic T lymphocytes were activated in mice after Con A induction, while CyA suppressed the activation of T cells, especially CD4^+^ T cells. The experiments suggested that CyA has immunosuppressive activity, which may contribute to its anti-inflammatory effect.

### 2.3. CyA Attenuates the Expression of IL-1β and ICAM-1 in the Liver of Con A-Injured Mice

**Figure 3:**
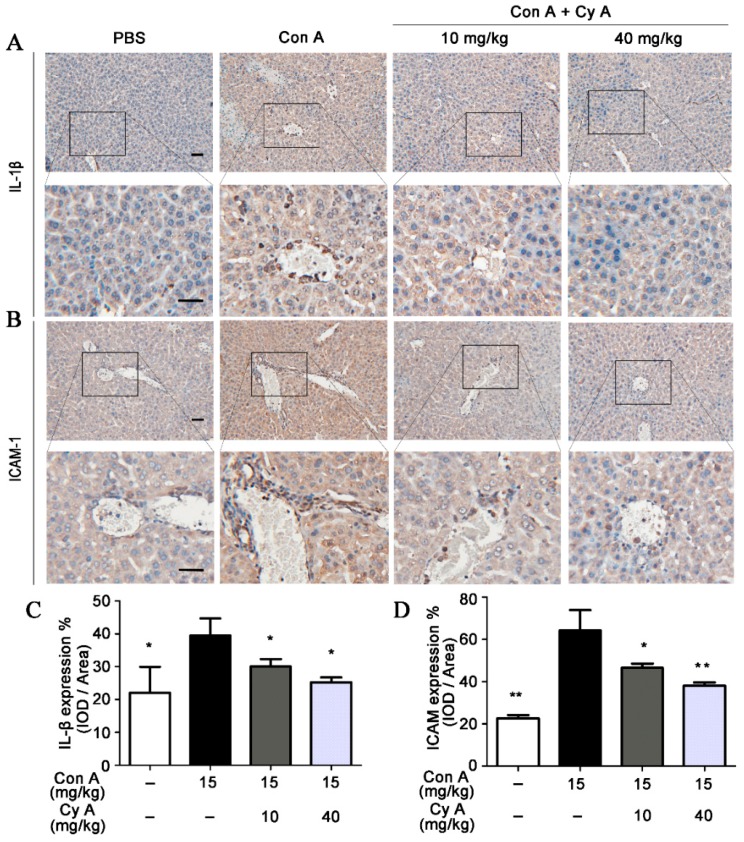
CyA attenuated the expression of IL-1β and ICAM-1 in the liver of Con A-injured mice. Mice were treated in the same way as described in Figure 1. The liver was removed and sectioned, and the expressions of IL-1β and ICAM-1 were stained with anti-IL-1β and anti-ICAM-1 antibodies and assessed by IOD method. The results shown in (**A**,**B**) are representative immunohistochemical pictures for IL-1β and ICAM-1 (bar = 100 μm). The data in (**C**,**D**) are expressed as mean ± SD from the three independent experiments. * *p* < 0.05, ** *p* < 0.01, versus the model group.

It is generally believed that inflammation is the key event of AIH, and lymphocyte recruitment to inflammatory areas is the characteristic of this event [26]. Intercellular adhesion molecule (ICAM)-1 is one of the important adhesion molecules between leukocytes and endothelial cells. It plays an essential role in mediating lymphocyte adhesion to vascular endothelial cells and inducing lymphocyte migration to inflammatory areas [27,28]. Pro-inflammatory cytokine IL-1β can increase ICAM-1 expression by activating related intracellular signaling pathways [29]. Therefore, the distributions and expressions of IL-1β and ICAM-1 in liver tissue of mice were analyzed by immunohistochemical and IOD semi-quantitative methods. As shown in Figure 3A,C, the expression of IL-1β was increased in Con A-stimulated liver tissue (*p* < 0.05, vs. the control), while the elevated IL-1β level was decreased by CyA treatment at doses of 10 and 40 mg/kg (*p* < 0.05 for both doses). Correspondingly, a significant expression (*p* < 0.01) of ICAM-1 particularly in the portal area was observed in Con A-injured liver tissue (Figure 3B,D). Compared with the model group, the expression of ICAM-1 in the CyA treatment groups was significantly inhibited (*p* < 0.05/0.01 for 10/40 mg/kg). The results suggested that CyA might inhibit Con A-induced liver inflammation by lowering elevated levels of IL-1β and ICAM-1.

### 2.4. CyA Alleviates Con A-Induced Liver Injury in Mice

**Figure 4:**
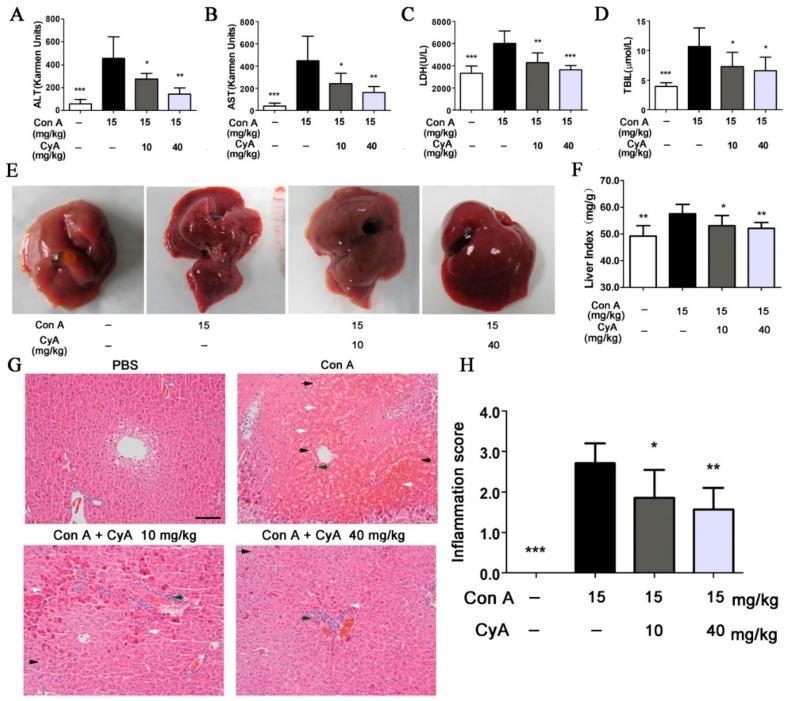
*CyA inhibited Con A-induced liver injury in mice*. Mice were treated in the same way as described in Figure 1. The liver injury was assessed by (**A**–**D**) the levels of ALT, AST, LDH and TBil in mouse serum (*n* = 5), also assessed by (**E**) morphology, (**F**) liver index (*n* = 5), (**G**) H&E stained liver section (bar = 100 μm) and (**H**) inflammation score (*n* = 5). White arrow indicates hepatocyte necrosis or apoptosis; black arrow indicates erythrocyte diapedesis; green arrow indicates lymphocyte infiltration. Data were expressed as mean ± SD. * *p* < 0.05, ** *p* < 0.01 and *** *p* < 0.001, versus the model group.

To assess the degree of liver injury in mice, the common biochemical indexes including ALT (alanine transaminase), AST (aspartate transaminase), LDH (lactic dehydrogenase) and TBil (total bilirubin) in serum of mice were tested. The levels of ALT, AST, LDH and TBil in Con A-injured mice were increased dramatically (*p* < 0.001 for all, vs. the control), while CyA treatment could significantly decrease the activities of ALT (*p* < 0.05/0.01 for 10/40 mg/kg), AST (*p* < 0.05/0.01 for 10/40 mg/kg), LDH (*p* < 0.01/0.001 for 10/40 mg/kg) and the level of TBil (*p* < 0.05 for both doses) (Figure 4A–D). Moreover, morphological and histopathological changes of liver in mice were also observed and analyzed. The liver of mice in the control group was normal in size and color, with smooth and lustrous surface and soft texture (Figure 4E). However, the liver in Con A-injured mice was enlarged and congestive, with darker color, scattered with bleeding spots and lesions on the surface. Treatment with CyA could improve the above morphological changes in model mice and obviously decreased the levels of liver index (*p* < 0.05/0.01 for 10/40 mg/kg) (Figure 4E–F). Histopathological examination further confirmed Con A-induced liver injury, which was characterized by sinusoidal congestion, hepatocyte degeneration, bridging necrosis and obvious inflammatory cell infiltration (Figure 4G). CyA could significantly reduce sinusoidal congestion and hepatic parenchymal cell necrosis at 10 mg/kg, especially at 40 mg/kg. (Figure 4G). Semi-quantitative evaluation showed that CyA also significantly reduced the inflammation scores at both doses (*p* < 0.05/0.01 for 10/40 mg/kg) (Figure 4H). These results indicated that CyA could effectively inhibit Con A-induced hepatitis in mice and might be a promising potential therapy for AIH.

### 2.5. CyA Protects Hepatocytes from Con A-Induced Apoptosis

**Figure 5:**
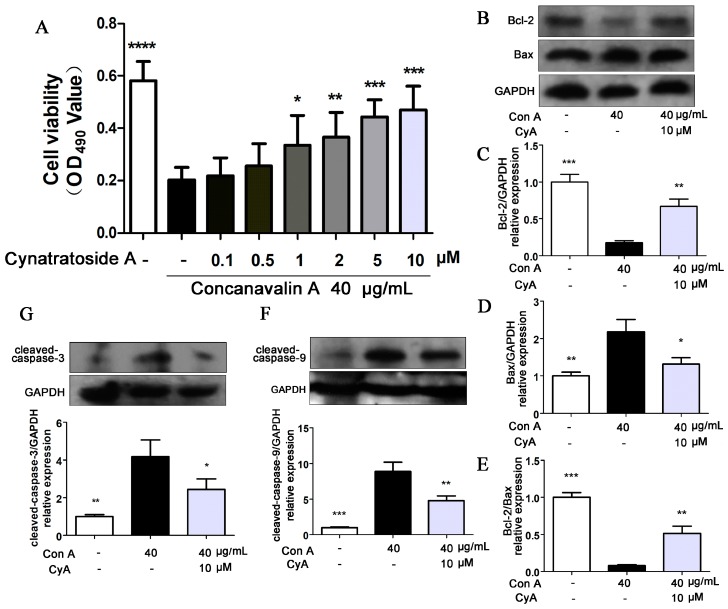
CyA protected L-02 hepatocytes from Con A-induced apoptosis. L-02 cells were seeded in 96-well plates for 24 h and co-incubated with 40 μg/mL Con A and CyA in indicated concentration. After 24 h of exposure, (**A**) cell viability was measured by MTT assay (*n* = 5), and the expressions of (**B**–**E**) Bcl-2/Bax and (**F**–**G**) caspase-9/3 were determined by western blot (*n* = 3). Data were expressed as mean ± SD. * *p* < 0.05, ** *p* < 0.01 and *** *p* < 0.001, versus the model group.

There is controversy over whether Con A has cytotoxic effects on hepatocytes [30,31,32]. Therefore, L-02 human hepatocytes were stimulated with 40 μg/mL of Con A and the cell viability was determined by MTT assay. Compared with the control group (Figure 5A), the proliferation of Con A-induced hepatocytes was significantly decreased (*p* = 0.000011), while CyA (0.1, 0.5, 1, 2, 5, 10 μM) could increase the cell proliferation in a concentration-dependent manner. This result indicated that high concentration of Con A can directly inhibit the proliferation of hepatocytes and CyA can protect hepatocytes from Con A-induced cytotoxicity. It was reported that hepatocyte apoptosis may be the mechanism of Con A-induced cell death [33,34]. Bcl-2 family proteins are molecular switch in apoptotic signaling pathways. Bcl-2 is an anti-apoptotic protein and Bax is a pro-apoptotic protein, and their ratio determines the survival or death of the cells [34]. Treatment of L-02 cells with Con A (40 μg/mL) significantly induced the expression of Bax (*p* < 0.01) and decreased the expression of Bcl-2 (*p* < 0.001) (Figure 5B–D). Thus, Con A significantly reduced the ratio of Bcl-2/Bax (*p* < 0.001) (Figure 5E), suggesting that Con A has a significant role in promoting hepatocyte apoptosis. Treatment with 10 μM CyA could reduce the expression of Bax (*p* < 0.05) and increased the expression of Bcl-2 (*p* < 0.01) (Figure 5B–D), and enhance the ratio of Bcl-2/Bax (*p* < 0.01) (Figure 5E). Caspase family is the most important effector of apoptosis [35,36,37]. Among them, caspase-9 and caspase-3 are the initiator and executor of apoptosis, respectively. When the L-02 cells were treated with Con A for 24 h, the expressions of cleaved caspase-9 (*p* < 0.001) and caspase-3 (*p* < 0.01) were increased significantly (Figure 5F,G). Treatment with 10 μM CyA could reduce the levels of cleaved caspase-9 (*p* < 0.01) and caspase-3 (*p* < 0.05) (Figure 5F,G), suggesting that CyA inhibited the activations of the two caspase proteins. These results suggested that Con A may mediate hepatocyte apoptosis in a caspase-dependent manner, and CyA could inhibit hepatocyte apoptosis by enhancing the Bcl-2 level and blocking the activation of caspase-dependent signaling pathway. 

## 3. Discussion

**Figure 6:**
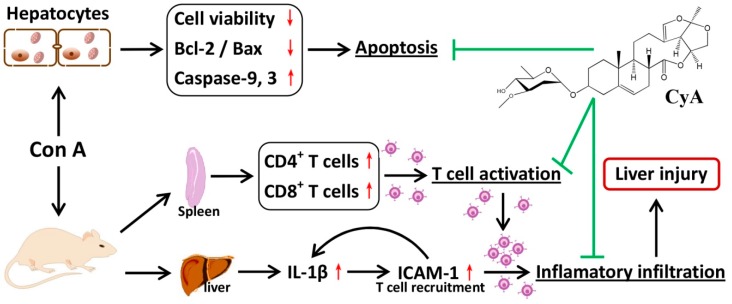
Chemical structure of CyA and a sketch of its effects on Con A-induced liver and hepatocyte injury.

CyA is one of the C_21_ steroidal glycosides with pregnane skeleton isolated from *C. atratum*. In the present study, the anti-AIH effect of CyA was investigated by using Con A-induced mouse and cell models. As summarized in Figure 6, CyA protects the liver from Con A-induced AIH by inhibiting the activation and adhesion of T lymphocytes and blocking hepatocyte apoptosis.

AIH is a progressive inflammatory disease mainly characterized by a large number of CD4^+^ T lymphocyte infiltration [7,8,9]. Con A-induced hepatitis is similar to human AIH in that the liver aggregates mainly CD4 + T cells, which mediates liver injury [20,21]. After intravenous injection, Con A binds specifically to the mannose gland in the surface of sinusoid endothelial cells (SECs), leading to the destruction of SECs, thus facilitating the binding of Con A to the Kupffer cells (KCs) [20]. After recognizing the MHC (major histocompatibility complex) class II and T cell receptor of KCs modified by Con A, CD4^+^ T cells are activated [20]. Therefore, the specific binding of Con A with SECs activates CD4^+^ T cells and recruits them from the blood to liver tissue. The large increase of lymphocyte homing to liver results in the continuous proliferation and differentiation of CD4^+^ T cells, which leads to the enlargement and structural disorder of spleen (Figure 1). CyA can improve the pathological changes of spleen and reduce the number of T cells by inhibiting T lymphocyte proliferation and activation in the spleen (Figure 2). However, the inhibition mechanism of CyA on the proliferation and activation of CD4^+^ T cells is not clear and needs further study.

Peripherally activated T lymphocytes and monocytes/macrophages are recruited to the inflammatory site of the liver, which is the precondition for promoting and sustaining the liver immune response [38]. In this process, the interactions among cell adhesion molecules, chemokines and their receptors are important steps [38]. Specifically, high expression of ICAM-1 in SECs is required for the recruitment, adhesion and infiltration of CD4^+^ T cells in Con A-mediated immune liver injury [39]. ICAM-1 is generally low expressed in cells, but inflammatory cytokines such as IL-1β can up-regulate the expression of ICAM-1 [40]. Increased ICAM-1 promotes immune cells to secrete more IL-1β, forming a positive feedback [41]. After Con A stimulation, the expressions of IL-1β and ICAM-1 in the liver tissue, especially in the portal area, of the model group were increased significantly, while their contents in CyA treated groups were decreased significantly (Figure 3). As a result, the liver injury induced by Con A was also significantly alleviated (Figure 4). These results suggest that the hepatoprotective effect of CyA involves reducing recruitment and adhesion of lymphocytes by inhibiting the secretion of IL-1β by KCs and the expression of ICAM-1 in SECs. However, the secretion of IL-1β by KCs is one of the upstream events in Con A-induced liver injury, and CyA can inhibit the secretion of IL-1β. It is suggested that CyA may inhibit Con A-induced T lymphocyte infiltration by blocking the binding of Con A to the surface receptors of SECs or KCs. This needs further study.

There are different opinions on whether Con A has direct hepatotoxicity [30,31,32]. Our in vitro experiments showed that Con A had direct hepatotoxicity, while CyA protected cells from toxicity. Bcl-2/Bax is the main regulator of mitochondrial apoptotic pathway [42,43]. Con A could induce hepatocyte apoptosis by reducing the ratio of Bcl-2/Bax (Figure 5B–E), indicating that mitochondria apoptotic pathway plays a key role in Con A-induced hepatotoxicity. CyA could effectively block hepatocyte apoptosis by regulating mitochondrial apoptotic pathway. However, it is not clear whether CyA acts on the upstream or the downstream of the mitochondrial signaling pathway.

## 4. Materials and Methods

### 4.1. Chemicals and Reagents

Con A was purchased from Sigma Aldrich (St. Louis, MO, USA). CyA was isolated from the root of *C. atratum* Bunge in our laboratory with a purity ≥ 98% (HPLC) [15]. Antibodies for IL-1β, ICAM-1, Bcl-2, Bax, caspases-3 and -9 were purchased from Wanlei Bio Co., Ltd. (Shenyang, China). Antibodies GAPDH and Goat anti-rabbit IgG-HRP were purchased from Bioworld Technology Co., Ltd. (St Louis Park, MN, USA). Enhanced chemiluminescent (ECL) plus reagent kit was purchased from Beyotime Institute of Biotechnology (Shanghai, China). All the other chemicals were commercial products of analytical or reagent grade.

### 4.2. Animals

Female ICR mice (18–24 g) were obtained from Comparative Medicine Center of Yangzhou University (Yangzhou, China). The animals were kept in a constant temperature (24 ± 2 °C) and relative humidity (50 ± 10%) with a 12 h light/dark cycle, and having free access to food and water. All animal experiments were executed in accordance with the Guide for the Care and Use of Laboratory Animals and the animal experiment protocols were approved by the Institutional Ethical Committee of China Pharmaceutical University, Nanjing, China (SCXK (Su) 2012-0004). Before the experiments, mice were fed for one week to adapt to the environment.

### 4.3. Con A-Induced AIH Model and Drug Treatment Protocols in Mice

Weight-matched mice were randomly assigned to four groups, i.e., the control, model and two drug groups. Con A (15 mg/kg, dissolved in phosphate-buffered saline, PBS) was injected to the mice through tail vein, and an equivalent volume of PBS was for the mice in the control group. CyA (10 and 40 mg/kg, suspended with 0.5% CMC-Na) was orally administrated to the mice 8 h before and 1 h after Con A treatment, and the mice in the control and model groups received an equivalent volume of 0.5% CMC-Na. The mice were sacrificed 16 h after Con A injection by excessive anesthesia with 6% chloral hydrate (10 mL/kg) to collect blood, liver and spleen. The serum was obtained by centrifuging the blood and stored in a refrigerator at −20 °C. After weighing, a part of the liver and spleen tissue were fixed in 10% formalin over 24 h and embedded in paraffin for histopathological and immunohistochemical examination, and the remaining part was quickly frozen in liquid nitrogen and stored at −80 °C.

### 4.4. Detection of Biochemical Indexes

The activity or levels of ALT (alanine transaminase), AST (aspartate transaminase), LDH (lactic dehydrogenase) and TBil (total bilirubin) in mouse serum were determined by commercial kits (Jiancheng Bioengineering Institute, Nanjing, China) in accordance with the manufacturer’s instructions.

### 4.5. Histopathological and Immunohistochemical Examination

The histopathological and immunohistochemical examination was performed using light microscopy. For histopathological, spleen and liver tissue sections (5 μm) were prepared and stained with hematoxylin and eosin (H&E). Inflammatory (histopathological) scores of spleen were classified as 0 (normal, no primary follicle hyperplasia), 1 (mild, secondary follicle hyperplasia), 2 (moderate, pathological changes of white pulp) and 3 (severe, necrosis). Inflammatory scores of liver were classified as 0 (normal, no or slight inflammation and no necrosis), 1 (slight, inflammation in portal area but no necrosis); 2 (mild, focal necrosis or eosinophilic bodies); 3 (moderate, severe focal necrosis) and 4 (severe, diffuse bridging necrosis). All sections were examined and assessed by professionals under light microscopy.

For immunohistochemical, liver tissue sections (5 μm) were prepared and stained with anti-IL-1β (1:100) and anti-ICAM-1 (1:100) antibodies. The expressions of IL-1β and ICAM-1 proteins were semi-quantified by integrated optical density (IOD) of at least three sections each with 5 microscopic fields using Image-Pro Plus image analysis software (version 7.0, Olympus, Tokyo, Japan).

### 4.6. Flow Cytometry Analysis of CD4^+^ and CD8^+^ Cells

The mouse splenic suspension was obtained by forcing the spleen through a 200-mesh stainless steel mesh and prepared in PBS. The suspension was centrifuged for 3 min at 4 °C with 900 rpm and then the Tris-NH_4_Cl red cell lysis solution was added to remove red blood cells. The solution was centrifuged again and the supernatant was discarded. After gently washed with PBS, the splenic cell pellets were collected. The isolated splenocytes were suspended in sterile PBS and stained with APC anti-mouse CD3ε, FITC anti-mouse CD4 or PE anti-mouse CD8a antibodies (BioLegend Inc, San Diego, CA, USA) for 30 min at 4 °C in the dark, washed with PBS and detected by MACSQuant™ flow cytometer (Miltenyi Biotec Co. Ltd., Bergisch Gladbach, Germany). The data were analyzed with FlowJo software (FlowJo VX, Tree Star Inc., Ashland, OR, USA).

### 4.7. Cell Culture and Treatment

L-02 cells, a normal human liver cell line from the Cell Bank of the Chinese Academy of Sciences (Shanghai, China), were grown in RPMI-1640 medium supplemented with 10% FBS, 100 U/mL penicillin and 100 μg/mL streptomycin and incubated at 37 °C in a humidified atmosphere (5% CO_2_). The medium was renewed every 2 days until the cells were grown to confluence. For experiments, L-02 cells were seeded in 6-well plates at 2 × 10^5^ cells/mL for 24 h. The cells were treatment with 40 μg/mL Con A and different concentrations of CyA for 24 h. The cell supernatant was collected for use.

### 4.8. Determination of Cell Viability

Cell viability was measured by MTT assay. Briefly, the cells were seeded in 96-well plates at 5 × 10^4^ cells/per well for 24 h. The cells were incubated with 40 μg/mL Con A and different concentrations of CyA for 24 h. MTT solution with a finally concentration of 0.5 mg/mL was added and the cells were incubated for 4 h. The insoluble formazan was collected and dissolved in DMSO and measured with a microplate reader (BioTek Instruments, Inc., Winooski, VT, USA).

### 4.9. Western Blot

Total proteins of the cells were extracted with 100 μL of ice-cold lysate buffer (Beyotime, Shanghai, China) containing 1% phenylmethanesulfonyl fluoride and centrifuged at 12,000 g for 10 min. Protein concentrations were determined with a BCA Protein Assay Kit (Beyotime, Shanghai, China). After boiling for 10 min, the extracted protein was loaded at 50 μg/per lane and fractionated on SDS-PAGE gel, electrophoretically transferred to a polyvinylidene difluoride membrane (Millipore, Billerica, MA, USA). With the primary antibody incubation overnight and the secondary antibody incubation at 25 °C for 2 h, the bands were detected by super ELC detection reagent (Beyotime, Shanghai, China). Images were evaluated using Image-Pro Plus software (version 7.0, Olympus, Tokyo, Japan).

### 4.10. Statistical Analysis

Data were expressed as the mean ± SD (standard deviation) from at least three independent experiments. Statistical analysis was performed by one-way analysis of variance (ANOVA) followed by Student’s two-tailed *t*-test (GraphPad Software, version 6). Value of *p* < 0.05 was considered to be statistically significant.

## 5. Conclusions

Our study suggested that CyA, a C_21_ steroidal glycoside from *C. atratum*, protects Con A-induced immune liver injury through inhibiting the activation and adhesion of T lymphocytes mediated by IL-1β and ICAM-1 and blocking hepatocyte apoptosis mediated by mitochondria apoptotic pathway. CyA may be a promising potential therapeutic agent for the treatment of AIH. It may also be an important lead compound for the development of derivatives with better efficacy.

## Abbreviations

AIH	autoimmune hepatitis
ALT	alanine transaminase
AST	aspartate transaminase
Bax	BCL2-associated X protein
Bcl-2	B-cell lymphoma-2
CC_50_	median cytotoxic concentration
Con A	concanavalin A
CyA	cynatratoside A
IC_50_	half maximal inhibitory concentration
ICAM-1	intercellular adhesion molecule-1
IOD	integrated optical density
IL-1β	interleukin-1β
KCs	Kupffer cells
LDH	lactic dehydrogenase
MHC	major histocompatibility complex
MTT	methyl thiazolyl tetrazolium
SECs	sinusoid endothelial cells
TBil	total bilirubin
